# Sumoylation and transcription regulation at nuclear pores

**DOI:** 10.1007/s00412-014-0481-x

**Published:** 2014-08-30

**Authors:** Lorane Texari, Françoise Stutz

**Affiliations:** 1 Department of Cell Biology, iGE3, University of Geneva, Geneva, Switzerland; 2 Present Address: The Salk Institute for Biological Studies, La Jolla, CA USA

## Abstract

Increasing evidence indicates that besides promoters, enhancers, and epigenetic modifications, nuclear organization is another parameter contributing to optimal control of gene expression. Although differences between species exist, the influence of gene positioning on expression seems to be a conserved feature from yeast to *Drosophila* and mammals. The nuclear periphery is one of the nuclear compartments implicated in gene regulation. It consists of the nuclear envelope (NE) and the nuclear pore complexes (NPC), which have distinct roles in the control of gene expression. The NPC has recently been shown to tether proteins involved in the sumoylation pathway. Here, we will focus on the importance of gene positioning and NPC-linked sumoylation/desumoylation in transcription regulation. We will mainly discuss observations made in the yeast *Saccharomyces cerevisiae* model system and highlight potential parallels in metazoan species.

## Nuclear organization of chromatin

It is well established that during interphase, mammalian chromosomes occupy distinct nuclear regions called chromosome territories (Hubner et al. 2013). In yeast, the concept of chromosome territories has also been proposed based on more frequent observation of intrachromosomal versus interchromosomal interactions (Rodley et al. 2009; Duan et al. 2010). In contrast to mammals, the subnuclear localization of yeast chromosomes is mainly driven by the localization of centromeres and telomeres at the nuclear periphery. Yeast centromeres are attached to the microtubule-organizing center, called the spindle pole body (SPB), which is inserted in the nuclear envelope opposite to the nucleolus, while telomeres are tethered at the nuclear periphery and clustered in four to six distinct foci (Gotta et al. 1996; Jin et al. 1998). The consequence of this nuclear organization is that chromosome positioning inside the nucleus is not random and that interchromosomal interactions may be governed by physical constraints such as chromosome length, centromere attachment to the SPB, and nuclear crowding (Schober et al. 2008; Therizols et al. 2010).

Because heterochromatin is mostly found at the nuclear periphery in higher eukaryotes, this compartment was associated with transcription repression. In these organisms, the inner nuclear membrane is lined with the nuclear lamina described to interact with chromatin directly or indirectly and to promote transcription repression (Kind and van Steensel 2010; Butin-Israeli et al. 2012; Towbin et al. 2013). Both in Drosophila and mammalian cells, the lamin-associated chromatin domains (LADs) correspond to as much as 40 % of the whole genome. LADs are mostly gene-poor regions, and genes located therein are five to ten times less active than genes outside of these domains. Consistent with their low expression levels, lamin-bound genes are rich in histone H3K27me3 and poor in histone H3K4me2 marks, two characteristics of repressive chromatin (Pickersgill et al. 2006; Guelen et al. 2008).

In agreement with the repressive nature of the nuclear periphery, early experiments performed in yeast revealed that artificial anchoring of the *GAL1* gene to the nuclear envelope promotes its silencing (Andrulis et al. 1998). Comparable approaches in mammalian cells showed that artificial targeting of a locus to the nuclear membrane leads to its repression in a mechanism dependent on histone deacetylases (HDACs) (Finlan et al. 2008; Reddy et al. 2008).

Hence, for many years, the nuclear periphery was thought to be a repressive compartment. However, several lines of evidence have recently emerged for the coexistence of a repressive and an activating compartment at the nuclear periphery corresponding, respectively, to the nuclear envelope and the nuclear pore complex. For example, artificial tethering of the yeast glucose repressed gene *HXK1* at the nuclear periphery *via* Esc1, a nuclear envelope protein implicated in NPC assembly, impacts on *HXK1* transcription in two opposing ways: It enhances *HXK1* repression in glucose but stimulates its expression under activating conditions, i.e., in the absence of glucose (Taddei et al. 2006). This study illustrates the dual role of the nuclear periphery in gene transcription regulation.

## Links between transcription and the NPC

The NPC is a large 60- to 125-MDa complex embedded in the nuclear envelope consisting of 30 different proteins, called nucleoporins or Nups, each present in multiple copies, as reviewed in D’Angelo and Hetzer (2008). There are about 200 NPCs per nucleus in yeast and up to 2,000 in mammalian cells. Despite differences in size and number of NPCs per nucleus, the overall architecture and NPC function are conserved from yeast to higher eukaryotes (Strambio-De-Castillia et al. 2010). Nuclear pores consist of a central core assembly containing the translocation channel framed by structures extending into the cytoplasm and the nucleus to form the cytoplasmic filaments and the nuclear basket, respectively. NPC basket proteins have been implicated in mRNP docking and quality control prior to mRNP nuclear exit (Strambio-De-Castillia et al. 2010).

Already in 1985, G. Blobel proposed the gene-gating model hypothesizing that active genes may relocate to nuclear pores to facilitate mRNA export (Blobel 1985). One of the first studies linking the NPC to gene activity was performed in yeast and showed that artificial targeting of Nup2 to a reporter gene promotes association of the locus with the pore resulting in a boundary that blocks heterochromatin spreading along the chromosome (Ishii et al. 2002). Subsequent genome-wide ChIP-on-chip analyses in yeast indicated that highly transcribed genes are more likely to interact with nucleoporins (Casolari et al. 2004). Furthermore, several inducible genes, including *HXK1*, *HSP104*, *INO1*, and the *GAL* genes, are enriched at the nuclear periphery when activated (Brickner and Walter 2004; Casolari et al. 2004; Dieppois et al. 2006; Taddei et al. 2006), and this relocalization was shown to be affected either directly or indirectly by mutations in some NPC basket nucleoporins, such as Nup1, Nup2, Nup60, Mlp1, and Mlp2 (Cabal et al. 2006; Dieppois et al. 2006; Dieppois and Stutz 2010; Texari et al. 2013). Although NPC localization is not essential for gene expression, artificial tethering of *INO1*, *GAL1*, or *HXK1* to the periphery promotes mRNA production (Brickner and Walter 2004; Taddei et al. 2006; Brickner et al. 2007). Thus, the localization of a gene to the NPC correlates with transcription; yet, the mechanisms by which highly active genes become more stably associated with the pore and more efficiently expressed in this context are still poorly understood.

**Fig. 1:**
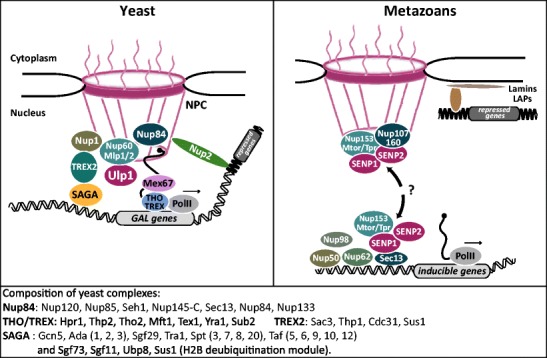
Gene to pore interactions in yeast are mediated by factors involved in transcription and mRNA biogenesis as well as NPC basket-associated proteins (*left*). The composition of the main yeast complexes involved in this process is indicated in the *box below the drawing*.f The SUMO protease Ulp1 and its mammalian counterparts SENP1 and SENP2 interact with homologous NPC components (*right*). While genes move to pores in yeast, nucleoporins are dynamic in metazoans and interact with target genes in the nucleoplasm. The factors conserved between yeast and metazoans are drawn with the same *color code*. See text for more details and references

The SAGA histone acetyltransferase coactivator complex is involved in the expression of a number of yeast-inducible genes (Holstege et al. 1998; Lee et al. 2000) and has been implicated in gene anchoring to the NPC. Indeed, the SAGA components Ada2 and Sus1 are required for *GAL* gene relocation to the NPC (Cabal et al. 2006; Luthra et al. 2007). Sus1 is also part of the NPC-associated TREX2 complex, involved in transcription-coupled mRNA export (Rodriguez-Navarro et al. 2004), and the TREX2 components Sac3 and Thp1 were shown to participate in NPC gene anchoring (Chekanova et al. 2008; Jani et al. 2014). The functional connection between the SAGA coactivator complex and TREX2 reinforces the gene-gating view, in which transcription at the NPC ensures optimal gene expression by facilitating rapid mRNA export through nuclear pores (Dieppois and Stutz 2010; Garcia-Oliver et al. 2012) (Fig. 1). The THO/TREX complex, recruited during transcription elongation by the RNA PolII machinery, has also been involved in coupling transcription and export, and we showed that early recruitment of the mRNA export receptor Mex67 by THO contributes to NPC tethering of the *GAL1-10-7* and *HSP104* genes (Dieppois et al. 2006). In Drosophila and *Caenorhabditis elegans*, SAGA and/or THO/TREX has also been implicated in NPC localization of heat shock genes, further supporting the evolutionary conservation of these basic processes (Kurshakova et al. 2007; Rohner et al. 2013).

Notably, additional studies proposed that the initial gene to pore association upon induction is independent of transcription or components of the SAGA complex (Schmid et al. 2006; Brickner et al. 2007), suggesting that relocalization to the NPC may occur before the gene is transcriptionally active and prior to SAGA recruitment. Accordingly, the *GAL1-10-7* locus is found at the NPC when cells are grown in raffinose, a condition under which the *GAL* genes are preinduced and RNA PolII is maintained in a poised state due to the masking of the Gal4 activation domain by the Gal80 repressor (Green et al. 2012; Jani et al. 2014).

Until now, a universal consensus sequence could not be found in the promoters of inducible genes that would explain their NPC localization. However, a sequence motif present in the promoter of *INO1* called GRSI (gene recruitment sequence I) was proposed to act as a DNA zip code that is both necessary and sufficient to target a gene to the NPC (Ahmed et al. 2010). DNA zip codes have also been implicated in interchromosomal clustering of genes sharing the same GRS at the nuclear periphery, although not necessarily at a single NPC (Brickner and Brickner 2012). The same laboratory identified additional zip codes called memory recruitment sequence (MRS) required for transcriptional memory, a process allowing faster gene reinduction after short-term repression and which requires the gene to stay at the periphery. Interestingly, an MRS is required for *INO1* memory as well as for incorporation of the histone variant H2A.Z and addition of histone H3K4me2 at the promoter upon repression (Light et al. 2010, 2013). While H2A.Z deposition is required to maintain the gene at the NPC after repression, both chromatin features participate in transcriptional memory (Brickner et al. 2007; Light et al. 2013). Gene maintenance at the periphery was also proposed to be important for *GAL1* gene transcription memory and to depend on NPC-associated Mlp1. Indeed, loss of Mlp1 prevents rapid reactivation of *GAL1* gene transcription following short-term repression in glucose (Tan-Wong et al. 2009).

Interestingly, transcription memory was also recently described in mammals for interferon gamma (IFN-γ)-inducible genes (Light et al. 2013). As in yeast, it depends on H3K4me2 and the binding of a specific nucleoporin (Nup98 in mammals vs Nup100 in yeast) to the target genes, but this interaction takes place away from the pores in mammals (Ptak et al. 2014; Sood and Brickner 2014).

Several nucleoporins have been shown to participate in transcription regulation in higher eukaryotes. Early studies in *Drosophila* suggested that Nup153 and Mtor, homologous to yeast Nup60 and Mlp1/2, respectively (Table 1), contribute to the two-fold upregulation of X-linked genes in male cells through interaction with the male-specific MSL complex, associated with the male X chromosome and essential for X chromosome dosage compensation (Mendjan et al. 2006). However, peripheral localization may not be essential for X chromosome regulation (Grimaud and Becker 2009; Vaquerizas et al. 2010). Three more recent studies performed in *Drosophila* using either DamID or ChIP-on-chip analyses showed that Nup153, Mtor, and several other nuclear basket nucleoporins including Nup50, Nup62, Nup98, and Sec13 associate with multiple genes, of which many are inducible, suggesting that these nucleoporins may play a role in transcription activation (Capelson et al. 2010; Kalverda et al. 2010; Vaquerizas et al. 2010). The identified targets are mostly developmental and cell cycle genes, indicating that nucleoporins in higher eukaryotes may primarily affect tightly regulated genes. Consistent with this idea, a recent study performed in mammalian cells showed that Nup210 is required for the conversion of embryonic stem cells into muscle or neuro-progenitors, suggesting an important role for this nucleoporin in the regulation of genes involved in cell differentiation (D’Angelo et al. 2012).

**Table 1:** List of yeast proteins discussed in the text

Yeast proteins	Sumoylated	Ulp1 target	Metazoan homologs	References for yeast proteins
Transcription repressors				
Cyc8/Ssn6	yes	yes	UTX/UTY	Panse et al. 2004; Wohlschlegel et al. 2004; Denison et al. 2005; Albuquerque et al. 2013; Texari et al. 2013
Tup1	yes	yes	Groucho (a)	Panse et al. 2004; Wohlschlegel et al. 2004; Denison et al. 2005; Wykoff and O’Shea 2005; Albuquerque et al. 2013; Texari et al. 2013
Transcription activators				
Gcn5 (SAGA)	yes	ND	GCN5/KAT2A/PCAF	Wohlschlegel et al. 2004; Sterner et al. 2006; Albuquerque et al. 2013
Ada2 (SAGA)	yes	ND	hADA2 (or TADA2A/B)	Wohlschlegel et al. 2004; Wykoff and O’Shea 2005
Spt7 (SAGA)	yes	ND	hSpt7 (or SUPT7L) (a)	Wohlschlegel et al. 2004; Wykoff and O’Shea 2005; Albuquerque et al. 2013
Cti6	yes	ND		Wohlschlegel et al. 2004; Albuquerque et al. 2013
Gcn4	yes	ND		Wohlschlegel et al. 2004; Rosonina et al. 2012
Snf1 (b)	yes	yes	AMPK (a)	Wohlschlegel et al. 2004; Denison et al. 2005; Simpson-Lavy and Johnston 2013
Histones				
H2A	yes	ND	H2A	Nathan et al. 2006
H2B	yes	ND	H2B	Nathan et al. 2006
H3	yes	ND	H3	Nathan et al. 2006
H4	yes	ND	H4	Nathan et al. 2006
H2A.Z	yes	ND	H2AZ (or H2AFZ)	Kalocsay et al. 2009
Chromatin modifiers				
Rpd3	ND	ND	HDAC1 (a) (b)	Wykoff and O’Shea 2005
Hda1	yes	ND	HDAC4 (a)	Panse et al. 2004; Wykoff and O’Shea 2005
NPC components				
Mlp1	yes	ND	Mtor/TPR	Denison et al. 2005; Wohlschlegel et al. 2004; Albuquerque et al. 2013
Mlp2 (b)	yes	ND	Mtor/TPR	Denison et al. 2005; Wohlschlegel et al. 2004; Albuquerque et al. 2013; Dargemont lab (pers. comm.)
Nup60 (b)	yes	yes	Nup153 (a)	Albuquerque et al. 2013; Dargemont lab (pers. comm.)
Nup2	yes	ND		Albuquerque et al. 2013

Metazoan counterparts shown to be sumoylated (a). Proteins that contain a demonstrated or proposed SIM domain (b). Note that some proteins indicated as sumoylated based on mass spectrometry analyses have not yet been confirmed by specific sumoylation assays and may represent non-sumoylated copurifying partners. See text for additional references

In contrast to yeast, transcription regulation mediated by nucleoporins in higher eukaryotes may occur mostly in the nucleoplasm rather than at the NPC (Fig. 1). Indeed, early iFRAP experiments have shown that in mammalian cells, a number of nucleoporins associated with the NPC nuclear basket are mobile and continuously exchange between the nuclear interior and the periphery (Rabut et al. 2004). Moreover, the nucleoporins involved in gene expression in *Drosophila* become associated with their targets inside the nucleoplasm, away from the nuclear periphery. Importantly, knockdown and overexpression of these nucleoporins, respectively, decrease and increase the expression of the target genes, strengthening the view that binding of these dynamic NPC components to the genes directly affects transcription (Capelson et al. 2010; Kalverda et al. 2010; Vaquerizas et al. 2010; Light et al. 2013; Ptak et al. 2014).

A popular model postulates that gene targeting to the NPC in yeast is not based on an active transport but relies on the continuous movement of genes within the nucleus and their attachment to the pore when they reach the nuclear periphery by passive diffusion (Dion and Gasser 2013). Nucleoporins and proteins bound to the pore may then stabilize this association. This mechanism requires changes at gene promoters increasing their affinity for the NPC. One possibility could be that the sumoylation state of promoter-bound proteins influences gene or promoter association with the pore. Indeed, recent studies have implicated sumoylation as a signal to target telomeres or damaged DNA to the nuclear periphery (Nagai et al. 2008; Ferreira et al. 2011). Importantly, many proteins involved in transcription regulation are sumoylated (Panse et al. 2004; Wohlschlegel et al. 2004; Zhao et al. 2004b; Zhou et al. 2004; Wykoff and O’Shea 2005; Albuquerque et al. 2013; Rouviere et al. 2013), and key factors involved in the sumoylation pathway are found in association with NPCs (Palancade and Doye 2008; Nagai et al. 2011). In addition, our recent studies suggest that the SUMO protease Ulp1, associated with the NPC, participates in *GAL1* gene relocalization to the pore (Texari et al. 2013).

## The SUMO pathway and nuclear pores

Sumoylation is a posttranslational modification consisting in the attachment of the small (10–11 kDa) evolutionarily conserved polypeptide SUMO (small ubiquitin-like modifier) on lysine residues. In *Saccharomyces cerevisiae*, SUMO is encoded by a single gene *SMT3*, while several genes code for several SUMO peptides (SUMO1, 2, 3, and 4) in higher eukaryotes (Geiss-Friedlander and Melchior 2007). SUMO has to be processed and activated before conjugation to its target proteins (Johnson 2004). SUMO processing is mediated by the SUMO protease Ulp1, which cleaves the last three amino acids after a GG motif. Before its addition to the target protein, yeast SUMO depends on an enzymatic cascade, which involves an E1 SUMO-activating enzyme (Uba2/Aos1 heterodimer), an E2 SUMO-conjugating enzyme (Ubc9), and in most cases an E3 ligase (Siz1, Siz2, Mms21, and Zip3). E3 ligases promote the attachment of SUMO to proteins and seem to confer target specificity, although E3 ligases present some redundancy (Palancade and Doye 2008). In yeast, removal of sumoylation is performed by two SUMO proteases: the essential protein Ulp1 and the nonessential protein Ulp2, which differ by their localization (Li and Hochstrasser 1999, 2000; Palancade and Doye 2008). The closest Ulp1 homologs in mammals are SENP1 and SENP2, while Ulp2 is homologous to SENP6 and SENP7, as reviewed in Hickey et al. (2012).

SUMO proteomics studies indicate that most sumoylated proteins are nuclear (Panse et al. 2004; Wohlschlegel et al. 2004; Zhao et al. 2004b; Zhou et al. 2004; Wykoff and O’Shea 2005; Albuquerque et al. 2013) and involved in numerous processes from chromosome segregation, DNA repair, and DNA replication, to nuclear transport, transcription, and regulation of telomere length (Melchior et al. 2003; Johnson 2004; Geiss-Friedlander and Melchior 2007; Torres-Rosell et al. 2007; Ferreira et al. 2011; Cremona et al. 2012; Bergink et al. 2013; Rouviere et al. 2013).

Sumoylation has been described to affect protein-protein or protein-nucleic acid interactions either through steric hindrance or by inducing conformational changes. Sumoylation can also regulate other posttranslational modifications such as ubiquitination or acetylation, as reviewed in Gareau and Lima (2010) and Rouviere et al. (2013). It was initially proposed that SUMO acts as an antagonist by competing with ubiquitination and thus counteracting degradation by the proteasome (Hoege et al. 2002; Verger et al. 2003). More recent reports showed that sumoylation can also promote ubiquitination by SUMO-targeted ubiquitin ligases (STUbLs), a process often coupled to degradation by the proteasome (Perry et al. 2008; Geoffroy and Hay 2009; Hickey et al. 2012; Simpson-Lavy and Johnston 2013). The recognition of sumoylated proteins by the yeast STUbL Slx5/Slx8 is mediated by SUMO-interacting motifs (SIM) present on both Slx5 and Slx8 (Simpson-Lavy and Johnston 2013; Sriramachandran and Dohmen 2014). Importantly, SIM domains are present in a number of proteins and promote interaction with sumoylated partners, thereby enhancing the assembly and stability of complexes composed of sumoylated and SIM-containing proteins, as reviewed in Garcia-Dominguez and Reyes (2009) and Sun and Hunter (2012). In higher eukaryotes, the formation of promyelocytic leukemia (PML) nuclear bodies depends on such interactions and contributes to gene regulation and genome stability by sequestration of sumoylated transcription regulators and factors involved in chromosome integrity (Hattersley et al. 2011; Hickey et al. 2012). Networks of SUMO-SIM interactions may also modulate the assembly of repressive chromatin complexes (Bernardi and Pandolfi 2007; Garcia-Dominguez and Reyes 2009; Hattersley et al. 2011; Hickey et al. 2012).

Notably, a number of enzymes implicated in the SUMO pathway are found in association with nuclear pores. In yeast, the Slx5/Slx8 STUbL copurifies with Nup84 (Nagai et al. 2008). Another important NPC-bound protein is the SUMO protease Ulp1 (Li and Hochstrasser 2003), implicated in DNA replication and repair, the formation and nuclear export of 60S ribosomal subunits as well as mRNA surveillance (Stelter and Ulrich 2003; Zhao et al. 2004a; Panse et al. 2006; Lewis et al. 2007; Palancade et al. 2007) and reviewed in Palancade and Doye (2008). Anchoring of Ulp1 at the pore is impaired in strains lacking Nup60, a component of the NPC nuclear basket. Nup60 is also implicated in the association of Mlp1/2 with the NPC, and removal of Mlp proteins nearly eliminates Ulp1 from the NPC (Zhao et al. 2004a). Moreover, mutations in Nup84 components affect the localization of Ulp1, indicating that this complex also participates in the binding of Ulp1 to the NPC (Palancade et al. 2007). Interestingly, these Ulp1 anchoring pathways appear conserved in mammals. Indeed, Nup153 and the Nup107/160 complexes, homologous, respectively, to yeast Nup60 and the Nup84 complex, contribute to NPC tethering of SENP1 and SENP2 (Hang and Dasso 2002; Zhang et al. 2002; Bailey and O’Hare 2004; Goeres et al. 2011; Chow et al. 2012). In addition, both Nup153 and Nup60 are sumoylated proteins (Chow et al. 2012) (Table 1). The pathway redundancy to keep Ulp1 at the pore and the conservation between species suggest that localization of Ulp1 at the NPC is of crucial importance.

## Ulp1: a player in the derepression of inducible genes at the NPC?

**Fig. 2:**
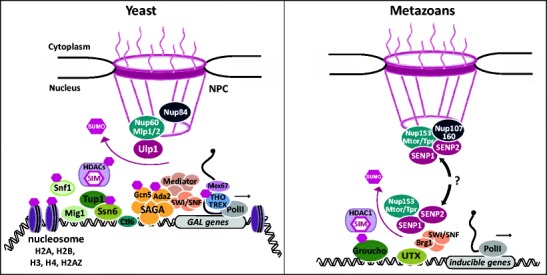
A speculative model is that the yeast NPC-associated SUMO protease Ulp1 (*left*) may desumoylate many target proteins involved in chromatin organization and transcription when active genes relocate to the pore, but also factors involved in DNA repair (not shown). In metazoans (*right*), SENP1 and SENP2 may move away from the NPC in association with dynamic pore components and desumoylate their specific targets within the nucleoplasm. The factors conserved between yeast and metazoans are drawn with the same *color code*

Our recent studies addressed the potential role of Ulp1 in transcription by examining the activation kinetics of the galactose-inducible *GAL1* gene. *GAL1* is fully repressed in glucose, and induction of *GAL1* by shifting cells from glucose to galactose is a very slow process (several hours), which involves an initial derepression step in order to achieve optimal coactivator recruitment. Derepression consists of extensive chromatin remodeling and the dissociation of the repressor Mig1 from the promoter, a process induced by phosphorylation of Mig1 by the Snf1 kinase (Papamichos-Chronakis et al. 2004). The *GAL1* gene is in a preinduced state when cells are grown in raffinose; in this case, the addition of galactose results in fast activation of mRNA transcription (min), which mainly relies on the recruitment of the SAGA coactivator complex by the Gal4 activator (Papamichos-Chronakis et al. 2004). By using these two modes of induction, we recently showed that loss of Mlp1/2 negatively affects the activation step but increases *GAL1* derepression kinetics (Texari et al. 2013). Interestingly, a delocalized Ulp1 mutant lacking its NPC anchoring domain showed the same accelerated derepression of *GAL1* but no effect on the activation step. These data suggest that the fast derepression phenotype in the absence of Mlp1 and Mlp2 is linked to the displacement of Ulp1 into the nucleoplasm, where this SUMO protease may desumoylate specific targets at the wrong time and place, resulting in altered *GAL1* derepression kinetics. This model was further validated by artificially anchoring *GAL1* to the NPC or conversely by tethering Ulp1 to the *GAL1* gene. Together, these observations support the view that Ulp1-dependent desumoylation of gene-bound targets may positively influence transcription kinetics in the context of the NPC (Fig. 2).

While our work suggests that Ulp1 enhances transcription at the NPC by facilitating derepression (Texari et al. 2013), other studies proposed that the NPC may participate in activation (Menon et al. 2005; Sarma et al. 2007) but also in gene repression (Sarma et al. 2011; Green et al. 2012). Indeed, loss of Nup120 and Nup133 was reported to reduce Mig1 association with its target gene *SUC2* resulting in increased *SUC2* mRNA expression (Sarma et al. 2011; Green et al. 2012). Furthermore, loss of the nucleoporin Nup1 leads to faster *GAL1* mRNA accumulation, similarly suggesting that the NPC has a negative effect on *GAL1* transcription and facilitates repression in glucose (Green et al. 2012). One view to reconcile these results with our observations would be that the nucleoporins implicated in repression act upstream of Ulp1 and contribute, directly or indirectly, to the maintenance of this protein at the NPC. Consistent with this idea, both Nup120 and Nup133 are components of the Nup84 complex, required for the tethering of Ulp1 at NPCs (Palancade et al. 2007). Interestingly, the nuclear basket component Nup2 has also recently been implicated in the maintenance of Ulp1 at the NPC (Srikumar et al. 2013), and deletion of both *NUP1* and *NUP2* is synthetic lethal, suggesting a redundant role of these nucleoporins (Loeb et al. 1993). We could therefore speculate that deletion of *NUP1* may increase the amounts of Ulp1 in the nucleoplasm and thus enhance the kinetics of *GAL1* derepression as observed in our study (Texari et al. 2013). It has been shown that the mammalian homologs of Ulp1, SENP1 and SENP2, interact with Nup153 (homologous to yeast Nup60) through a specific FG-independent sequence of 17 amino acids (Chow et al. 2012). Interestingly, this specific sequence has been found at the tail of the yeast Nup1 protein (Sistla et al. 2007), reinforcing the idea that Nup1 may interact with Ulp1.

As mentioned above, several metazoan nucleoporins, including Nup98, Nup153, Mtor (homologous to yeast Mlp1/2), and Sec13 (component of Nup107/160 complex homologous to yeast Nup84C), have been implicated in transcription regulation (Capelson et al. 2010; Kalverda et al. 2010; Vaquerizas et al. 2010; Light et al. 2013). In addition, both Nup153 and Nup107/160 are able to interact with SENP1 and SENP2 in mammalian cells (Goeres et al. 2011; Chow et al. 2012). Moreover, SENP2 was proposed to positively influence transcription activation in mammals (Best et al. 2002; Ross et al. 2002). Combining these observations with our findings, we could speculate that in higher eukaryotes, nucleoporins regulate gene expression through interaction with SENP1 and SENP2. However, in metazoans, transcription regulation by this nucleoporin-dependent mechanism may take place mostly within the nucleoplasm and not at the NPC (Figs. 1 and 2).

Yet, this view is probably oversimplified. Indeed, Ulp1, SENP1, and SENP2 associate with NPCs through similar mechanisms involving either direct or indirect interactions with nucleoporins. The association or dissociation of all these proteases from the NPC may therefore be subject to similar modes of regulation to control localization and function. While we did not observe any change in Ulp1 localization when shifting cells from glucose to galactose (L. Texari, unpublished data), Ulp1 has been shown to relocate to the septin ring in mitosis to regulate septin desumoylation (Makhnevych et al. 2007), as well as to dissociate from NPCs and relocate to the nucleolus in response to ethanol stress for still unknown reasons (Sydorskyy et al. 2010). One could therefore imagine that Ulp1, as proposed for SENP1 and SENP2, could be mobilized to the nucleoplasm to control gene expression under specific conditions. On the other hand, it may be possible that “gene gating” also occurs in large eukaryotes and vertebrate cells, with NPC-associated SENP1 and SENP2 controlling transcription of some genes at the nuclear membrane similar to Ulp1. This could be the case for X-linked dosage compensated and heat shock genes in *Drosophila melanogaster* (Mendjan et al. 2006; Kurshakova et al. 2007; Vaquerizas et al. 2010), heat shock genes in *C. elegans* (Rohner et al. 2013), as well as the mammalian IFN-gamma (Hewitt et al. 2004) or β-globin (Ragoczy et al. 2006) loci, which associate with the nuclear periphery either constitutively or transiently upon transcription activation during the differentiation process.

## Potential Ulp1 targets involved in transcription at the NPC

A majority of factors modified by SUMO are nuclear proteins involved in a variety of processes. In particular, sumoylation of transcription regulators has often been linked to transcription repression (Garcia-Dominguez and Reyes 2009) (Table 1 and Fig. 2).

The yeast Ssn6-Tup1 corepressor complex is involved in the repression of numerous yeast genes, such as cell cycle-regulated genes, and genes expressed under different environmental stress conditions including poor carbon source (Smith and Johnson 2000; Zhang and Reese 2004). Thus, Ssn6-Tup1 represses a number of genes in glucose, including the galactose-inducible genes. The recruitment of Ssn6-Tup1 to *GAL* genes was initially described to depend on the glucose repressor Mig1; however, more recent studies indicate that Ssn6-Tup1 is bound to the *GAL* promoter both in repressive and activating conditions (Papamichos-Chronakis et al. 2002). Under repressive conditions, the complex was proposed to interact with the histone deacetylases Rpd3 and Hos2 (class I HDACs) as well as Hda1 (class II HDAC), resulting in H3 (Wu et al. 2001; Davie et al. 2002, 2003; Davie and Dent 2004) and H2B deacetylation (Wu et al. 2001). While Ssn6 interacts with DNA-binding proteins, Tup1 was proposed to function in HDAC recruitment (Zhang and Reese 2004). Consistently, deletion of *HDA1* leads to H3 and H2B hyperacetylation at Ssn6-Tup1 target promoters (Wu et al. 2001; Wong and Struhl 2011) and upregulation of genes repressed by Tup1 (Robyr et al. 2002). In contrast, under activation conditions, Ssn6-Tup1 contributes to the recruitment of SWI/SNF and the SAGA coactivator complex, facilitating histone acetylation by Gcn5 (Papamichos-Chronakis et al. 2002; Proft and Struhl 2002). Thus, Ssn6-Tup1 either decreases or promotes histone acetylation depending on the growth condition. One possibility is that the Ssn6-Tup1 complex switches from a repressive to an activating state upon galactose induction by undergoing conformational changes that modify its affinity for various partners.

Importantly, both Ssn6 and Tup1 are sumolyated proteins (Panse et al. 2004; Wohlschlegel et al. 2004; Wykoff and O’Shea 2005; Albuquerque et al. 2013), and we showed that Ulp1 delocalization from the NPC decreases Ssn6 sumoylation (Texari et al. 2013). Moreover, we observed that absence of Ssn6 sumoylation correlates with accelerated *GAL1* transcript accumulation and showed that rescue of sumoylation restores normal *GAL1* mRNA levels (Texari et al. 2013). Thus, sumoylation may participate in the switch of Ssn6-Tup1 from a repressive to an activating state. The NPC-tethered SUMO protease Ulp1 may therefore contribute to optimal transcription activation kinetics at the pore via desumoylation of transcription regulators associated with genes relocating to the NPC upon induction. One possibility is that Ssn6 desumoylation promotes the putative conformational change that facilitates interaction of DNA-binding proteins with coactivators (Fig. 2). More specifically, Ssn6 has been shown to recruit Cti6 to the *GAL1* promoter. Cti6 was proposed to relieve transcriptional repression by mediating the interaction between Ssn6-Tup1 and the SAGA component Gcn5. Indeed, loss of Cti6 prevents interaction between SAGA and Ssn6-Tup1 and impairs Gcn5 occupancy at the *GAL1* promoter (Papamichos-Chronakis et al. 2002). Accordingly, a recent study proposed that repression by Ssn6-Tup1 involves masking of the activation domain of activators thereby blocking the recruitment of coactivators such as SWI/SNF, SAGA, and mediator complexes (Wong and Struhl 2011). In light of these results, sumoylated Ssn6 may mask the domain of Cti6 involved in SAGA recruitment. Desumolyation of Ssn6 could change the conformation of the Ssn6-Tup1-Cti6 complex and allow interaction of Cti6 with the SAGA complex. This model predicts that the interaction between Cti6 and the SAGA component Gcn5 should be increased in mutants in which Ssn6 sumoylation is affected.

Interestingly, UTX, the mammalian homolog of Ssn6 (Smith and Johnson 2000), is recruited to cardiac specific enhancers and proposed to activate cardiac genes by recruiting the SWI/SNF component Brg1 during cardiac development (Lee et al. 2012). The authors proposed that UTX, which is also a H3K27 demethylase, plays a role in the transition from repressed to active chromatin during heart development. These observations suggest that the Ssn6 OFF/ON switch may be a conserved mechanism and that sumoylation may regulate the activity of UTX also in higher eukaryotes (Fig. 2).

Because the effect of the non-sumoylated Ssn6 mutant on *GAL1* activation kinetics is modest (Texari et al. 2013), Ulp1 is likely to desumoylate additional targets at the pore, resulting in optimal activation kinetics in this context. Consistently, numerous transcription activators, repressors, and histones are sumoylated (Panse et al. 2004; Wohlschlegel et al. 2004; Wykoff and O’Shea 2005; Nathan et al. 2006; Albuquerque et al. 2013; Rouviere et al. 2013) (Table 1). Among those, the activity of the Snf1 kinase, implicated in Mig1 phosphorylation, is negatively regulated by sumoylation (Simpson-Lavy and Johnston 2013). It is therefore possible that the described relocation of Snf1 to the nuclear periphery upon glucose depletion (Sarma et al. 2007) favors its desumoylation by Ulp1 triggering Mig1 phosphorylation and dissociation from repressed genes at the NPC (Fig. 2).

Besides transcription factors, the sumoylation of chromatin itself may contribute to gene repression. In yeast, all four core histones are sumoylated, and subtelomeric regions are more sumoylated than internal chromosome regions (Nathan et al. 2006). This study also showed that mutations in H2B leading to decreased sumoylation correlate with increased *GAL1* mRNA levels, and conversely that fusing SUMO to H2B decreases *GAL1* mRNA levels, indicating that sumoylation of histone H2B represses *GAL1* transcription. Furthermore, H2B acetylation and ubiquitination specific to actively transcribed genes negatively correlate with H2B sumoylation, suggesting that sumoylation competes with these modifications.

Interestingly, the histone variant H2A.Z is also sumoylated, and H2A.Z sumoylation participates in the repositioning of persistant DNA double-strand breaks at the NPC (Kalocsay et al. 2009). Moreover, H2A.Z has been implicated in transcriptional memory of *GAL1* as well as in the maintenance of *GAL1* at the NPC during repression (Brickner et al. 2007), suggesting a role for H2A.Z in DNA relocalization to the NPCs in different conditions. Finally, Tup1 was shown to facilitate H2A.Z deposition at the *GAL1* promoter upon repression, ensuring efficient recruitment of SAGA, mediator, and SWI/SNF and rapid activation (Gligoris et al. 2007; Lemieux et al. 2008). In light of these observations, one could speculate that H2A.Z sumoylation/desumoylation participates in gene localization as well as in the Ulp1-dependent derepression mechanism. Notably, like H2A.Z, Mlp1 has been involved in transcription memory by anchoring activated *GAL1* to the NPC and maintaining the locus in this location during short-term repression (Dieppois et al. 2006; Tan-Wong et al. 2009). Thus, H2A.Z and Mlp1 could act together in the same pathway. It would be interesting to define whether a non-sumoylated H2A.Z mutant affects *GAL1* gene anchoring and activation kinetics.

A number of chromatin-modifying enzymes are sumoylated, and the modification is usually linked to repression (Garcia-Dominguez and Reyes 2009). In mammalian cells, mutation of two lysines in HDAC1 decreases its sumoylation and alleviates its repressive activity (David et al. 2002). Interestingly, yeast Hda1 is also sumoylated (Table 1), and its desumoylation could therefore play a role in the derepression mechanism mediated by Ulp1. Moreover, the histone acetyltransferase Gcn5 is sumoylated and a potential target of Ulp1. We observed that the constitutive desumoylation of Gcn5 correlates with an increase in *GAL1* mRNA levels (L.T. unpublished data), further reinforcing the links between the sumoylation of a chromatin regulator and gene expression.

Our recent study shows that Ssn6 sumoylation is linked to *GAL1* repression, and as mentioned above, its partner Tup1 interacts with HDAC and acetyltransferase (HAT) (Wu et al. 2001; Papamichos-Chronakis et al. 2002; Davie et al. 2003; Davie and Dent 2004). Interestingly, in *Drosophila* cells, the sumoylation of the Tup1 homolog Groucho promotes the recruitment of HDAC1 through interaction with the SIM of this HDAC (Ahn et al. 2009). The sumoylation of Groucho has also been reported to enhance its repression activity. By analogy to the regulation of Groucho, we could speculate that in yeast, the Ssn6-Tup1 complex acts as repressor when sumoylated, in part because sumoylation may enhance the interaction with Hda1 or Rpd3 subunits, while Ssn6-Tup1 desumoylation would impair these interactions. The effect of sumoylation on the activity of a variety of mRNA biogenesis regulators in higher eukaryotes has been extensively reviewed elsewhere (Rouviere et al. 2013).

In conclusion, sumoylation may affect chromatin structure and function by diverse mechanisms. First, direct modification of chromatin by histone sumoylation may in turn affect histone acetylation and ubiquitination. Second, the sumoylation of HDAC and HAT proteins, which control the acetylation state of histones, may also contribute to the formation of repressive chromatin. Last but not least, the sumoylation state of proteins involved in the recruitment of HDACs and/or HATs at the promoter (such as Ssn6-Tup1 or Groucho) may also influence their interaction with these histone-modifying enzymes (Fig. 2).

## Conclusion

An emerging view is that a multitude of nuclear proteins are either sumoylated and/or contain SIM domains, which facilitate the formation of vast protein networks that contribute to the constitution of nuclear subdomains enriched in specific factors important for optimal regulation of gene expression (Hickey et al. 2012).

While we focused on the positive effect of desumoylation on gene expression, transcription factors such as Gcn4 become sumoylated during activation. In this case, sumoylation promotes ubiquitination and degradation of Gcn4 by the proteasome, favoring the rapid on-off switch of gene expression (Rosonina et al. 2012). These findings may nevertheless be consistent with our model since desumoylation by Ulp1 is expected to stabilize Gcn4 and hence to favor transcription. Thus, the sumoylation/desumoylation dynamics may be critical to fine-tune gene expression. Besides transcription initiation, sumoylation also regulates more downstream steps in mRNA biogenesis. Indeed, dynamic sumoylation/desumoylation of the THO component Hpr1, implicated in transcription elongation and mRNA export, was recently shown to protect a subset of stress-inducible transcripts from degradation by the nuclear exosome (Bretes et al. 2014).

Importantly, besides transcription and mRNA biogenesis, NPC-linked SUMO metabolism also contributes to genome stability. Indeed, both Ulp1 and the STUbL Slx5/Slx8 have been implicated in DNA repair and telomere maintenance (Nagai et al. 2011). These observations raise the question of whether highly expressed genes, potentially more prone to transcription-associated recombination, may relocate to NPCs to ensure genome integrity.
